# Therapeutic and vaccine strategies against SARS-CoV-2: past, present and future

**DOI:** 10.2217/fvl-2020-0137

**Published:** 2020-07-27

**Authors:** Mubasher Rehman, Isfahan Tauseef, Bibi Aalia, Sajid Hussain Shah, Muhammad Junaid, Kashif Syed Haleem

**Affiliations:** 1 ^1^Department of Microbiology, Hazara University, Mansehra 21300, Pakistan; 2 ^2^Pediatric Department, KMU Institute of Medical Science, Kohat, Pakistan; 3 ^3^Pediatric Department, Ayub Teaching Hospital, Abbottabad, Pakistan

**Keywords:** coronavirus, COVID-19, drugs, nCoV, SARS-CoV-2, vaccine

## Abstract

Severe acute respiratory syndrome coronavirus 2 (SARS-CoV-2) is the causative agent of coronavirus disease 2019. It was first reported in Wuhan, China and has since become a global health concern. Keeping in view, the magnitude of the problem, scientists around the globe are working to develop effective therapeutic strategies. This review focuses on previous findings regarding SARS-CoV, which may prove helpful in future research on SARS-CoV-2. In addition, it also highlights recent developments in medicine and biotechnology toward developing effective drugs and vaccines against SARS-CoV-2. This review will analyze available data on this topic and will help researchers develop new thoughts using information already available as a step toward developing novel therapeutic strategies against SARS-CoV-2.

Severe acute respiratory syndrome coronavirus 2 (SARS-CoV-2) also known as 2019 novel coronavirus (2019-nCoV) is the causative agent of COVID-19. COVID-19 is a severe respiratory disease which was initially noticed in Wuhan, China in December 2019 [1] and rapidly spread to more than 198 countries. The WHO declared COVID-19 as a global emergency on 30 January 2020 [2,3].

Over 3.1 million COVID-19 cases have been confirmed among which 224,172 deaths occurred till 1 May 2020 [3]. Especially in underdeveloped countries, these cases can be more than anticipated due to limited diagnostic facilities in many countries. Transmission of the virus can be human to human or animal to human [4]. The main clinical symptoms include dyspnoea, fever, dry cough, myalgia and difficulty in breathing while in severe cases, septic shock, kidney failure, pneumonia and even death was reported [2,5].

Initially, the virus spread across different countries, mainly because of travelling of the infected individual from China to other countries. Precautionary measures have been taken worldwide on airports and borders. Healthcare experts also advised that those who traveled from outside the country should be tested for COVID-19. If tested positive, the person must observe a 14-day quarantine. Some of the countries have not paid attention to precautionary measures especially for immigrants, which resulted in the global spread of the virus. The world is fighting continuously to combat COVID-19. Therefore, many companies and laboratories across the globe are working to develop different vaccines or drug to explore possible ways to treat this infection. This review will focus on the global prevalence and possible treatment strategies for COVID-19.

## Global prevalence

According to latest reports by the WHO, as of 1 May 2020, total cases of COVID-19 worldwide were 3,175,207 accounting for 224,172 deaths. In Africa, 26,663 confirmed cases and 973 deaths were reported, while in America, 1,291,917 confirmed cases and 69,087 deaths have been reported. In Eastern Mediterranean, 188,585 confirmed cases and 7598 deaths were confirmed. In Europe, 1,461,404 confirmed cases were reported among which 138,200 died because of COVID-19. In South East Asia, 57,088 cases have been confirmed among which 2174 died, while in the Western Pacific, 148,838 confirmed cases have been reported with 6127 deaths (Table 1) [3].

**Table 1.** Total number of cases worldwide as reported by WHO on 1 May 2020.

Continent	Reported cases	Death toll	Case fatality rate (%)
Globally	3,175,207	224,172	7.06%
Europe	1,461,404	138,200	9.45%
Americas	1,291,917	69,087	5.34%
Eastern Mediterranean	188,585	7598	4.02%
Western Pacific	148,838	6127	4.11%
South East Asia	57,088	2174	3.80%
Africa	26,663	973	3.64%

## Treatment strategies for COVID-19

Currently, no specific antiviral agent is available against SARS-CoV-2 infection, with clinically proven efficacy as in case of MERS-CoV and SARS-CoV [6,7]. To understand SARS-CoV replication, different animal models have been used and they showed severe infection symptoms. On the other hand, no pathogenesis was detected in case of MERS-CoV because of DDP4 receptor noncompatibility in mice [8]. For studying SARS-CoV-2 pathogenicity, SARS-CoV animal models can be used because SARS-CoV-2 is 80% similar to SARS-CoV. SARS-CoV-2 entry into host cells is thought to be similar to SARS-CoV, possibly through ACE2 cell receptor [9]. As per various genomic organization studies [10] and molecular mechanisms of SARS-CoV-2 pathogenesis [11], there are numerous targets, which can be used in different ways as therapeutic agents to inhibit the virus replication or to develop an intervention which may be effective against SARS-CoV-2. Various therapeutic (drugs), immunotherapeutic and Vaccination strategies against SARS-CoV-2 are summarized in Table 2 and subsequently discussed below.

**Table 2.** Therapeutic strategies against SARS-CoV-2.

Strategy	Methods	Mode of action	Examples/under trial drugs	Ref.
Receptor inhibition	a. ACE2 receptor inhibition b. TMPRSS2 inhibition	Inhibition of viral receptors on host cell by different compounds interfere with Spike or part of Spike refolding	Baricitinib, ruxolitinib, natural hesperidin, nafamostat mesylate, camostat mesylate and other antiviral drugs	[9,12–15]
Antiviral drugs	a. Antiviral drugs specifically designed for SARS-CoV-2 b. Antiviral drugs already used for other viral infections (such as HIV, hepatitis, influenza etc.) are now under clinical research against novel coronavirus	To identify the viral proteins and stop the replication of virus	Lopinavir/ritonavir, IFN-α, arbidol, favipiravir and darunavir etc., (all these drugs are already approved for other viral infections but now are under clinical trial against SARS-CoV-2). remdesivir, chloroquine and ivermectin are recently US FDA approved drugs for SARS-CoV-2 infection.	[16–18]
Immunotherapy	a. Complement system b. NK cell therapy c. IL-6 inhibitors d. mTOR inhibitors	Suppressing/inhibiting or enhancing the body immune response/s	C3a, C5a inhibitors, CYNK-001, tocilizumab, rapamycin	[19–24]
Vaccination	a. RNA vaccines b. DNA vaccines c. Recombinant protein vaccines d. Live attenuated vaccines e. Killed/inactivated vaccines f. Viral vector-based vaccines	Helps the immune system of the body to identify and to fight against infectious pathogens by providing acquired immunity (In all these vaccines target is S protein except live attenuated and killed vaccine for which the whole virion is the target)	Currently, more than 115 vaccines are developing for example: mRNA-1273 (RNA vaccine by Moderna Inc.), INO-4800 (DNA vaccine by Inovio Pharmaceuticals Inc.), ChAdOx1nCov-19 (killed/inactivated vaccine by Oxford University, AstraZeneca Plc.), Ad5-nCov (live attenuated vaccine by CanSino Biologics Inc.) and NVX-CoV2373 (recombinant protein vaccine by Novavax Inc and by Clover Biopharmaceuticals).	[25–27]
Other therapeutic options	a. Plasma therapy b. Medicinal plants c. Protease inhibitors d. Monoclonal antibody	Inhibit viral replication or to boost body immune response	*Artemisia annua* (medicinal plant), plasma from patient recovered from nCoV infection is used in plasma therapy. Protease inhibitors such as lopinavir, atazanavir and indinavir	[28–31]

NK: Natural killer; SARS-Cov-2: Severe acute respiratory syndrome coronavirus 2.

### Therapeutic strategies (drugs)

#### ACE2/TMPRSS2 receptor inhibition

Virus initially binds to a host cell through targeted receptors. Previous studies on SARS-CoV revealed that the primary targets of this virus are epithelial cells, macrophages and other cells in the lungs. All these cells have ACE2 which is used by SARS-CoV for attachment and entry [32–34]. Another study on immune response kinetics documented that cell spike (S) and hemagglutinin (H) are the anchoring proteins of SARS-CoV-2 with the help of which virus binds with host cells. S and H proteins are present on the surface of the virus and help in attachment with host cell receptor ACE2 and with sialic acid receptors respectively [33]. It was observed that increased infectivity of SARS-CoV-2 as compared with SARS-CoV is because of a furin-like cleavage site present on its S protein [35]. Along with furin pre-cleavage, TMPRSS2 also aids in the host cell entry and SARS-CoV-2 spike protein processing [36].

It was hypothesized that one way to stop SARS-CoV-2 infection is to saturate or block the ACE2 receptors or TMPRSS2 by using different molecules and antibiotic, which resultantly restrict virus from binding to the host cell and ultimately control the replication cycle [12]. Different drugs already approved for treatment of other conditions can be used to inhibit endocytosis mediated by ACE2. One such example is baricitinib used in treating rheumatoid arthritis. Similarly, Janus kinase could possibly be used for ACE2 inhibition [37]. Later this year, ruxolitinib, another Janus kinase inhibitor will be tested for COVID-19 treatment [38]. Another study reported that natural hesperidin can also be used to inhibit the ACE2 receptor [12]. Addition of a high concentration of ACE2 in a soluble form may reduce the entry of the virus into targeted host cells. Using different small molecules, which interfere with spike or part of spike refolding can possibly control the infection of virus [12,39]. Another study docked antiviral drugs with 21 possible targets based on their docking result. Findings revealed that among all possible dock targets, three of these namely Nsp3b, Nsp3c and E-channel are the suitable antiviral drugs. The effects of antiviral drugs on these targets need more research focus [12].

For TMPRSS2 inhibition, no clinical drug has yet been specifically tested in case of COVID-19 except for the camostat mesylate which stopped virus entry into the lung cells [36,37]. On the other hand, in case of other infections, clinical trials of nafamostat mesylate [40,41] and Camostat mesylate [36] have already been approved for TMPRSS2 inhibition. Anti-corona virus activity of imatinib has previously been reported as it inhibits the endosomal membrane and viral fusions [42]. Involvement of ACE2 receptors in facilitating viral entry into the cells makes them potential antiviral drug targets to control the entry of virus into the host cell.

#### Antiviral drugs

Antiviral drugs are designed in such a way that they identify the viral proteins and stop the replication of virus instead of killing them as antimicrobials do. By stopping the replication cycle, they tend to reduce the number of pathogens to a certain level where they are unable to induce pathogenesis and allow the body’s own immune response to neutralize the virus [43].

WHO, US FDA, European Medicines Agency (EMA) and the Chinese government and drug manufacturers have coordinated with different institutes and industrial scientists to develop antiviral drugs. The International Clinical Trials Registry Platform of the WHO approved hundreds of the clinical studies to carry out and test postinfection therapies for COVID-19 infections [16,44] with many antiviral drugs already used for treatments of other infections [17,18,45]. These drugs have previously been tested and used against HIV, malarial parasites, influenza viruses, hepatitis C virus etc. Some of the antiviral drugs, which have been tested *in vitro* as inhibitors of SARS-CoV-2 infection are: IFN-α, previously used to treat hepatitis infection and has shown to inhibit the replication of SARS-CoV [46]. Hence, this drug may possibly inhibit the replication of SARS-CoV2. *In vitro* as well as clinical studies have documented that Lopinavir/ritonavir, an HIV drug has anti coronavirus activity [47,48]. Antiviral drug arbidol which is used against influenza virus has also proven to be effective in inhibiting infection caused by SARS-CoV-2 [49]. Favipiravir and darunavir drugs which are used against novel influenza and HIV also showed anti-SARS-CoV activity [49,50].

According to one study, it was suggested that remdesivir and chloroquine can be used to treat COVID-19 infected patients because they showed promising effects *in vitro* [40,51]. Based on these findings, FDA approved the antiviral drug remdesivir for the treatment of laboratory-confirmed COVID-19 adults and children; however, no evidence exists about the safety and effectiveness of remdesivir [52]. Prevention is the utmost way to control infection.

Ivermectin, an antiparasitic drug has also been approved recently by the FDA against COVID-19 after successful inhibition of replication of SARS-CoV-2 *in vitro* [53].

**Figure 1.**
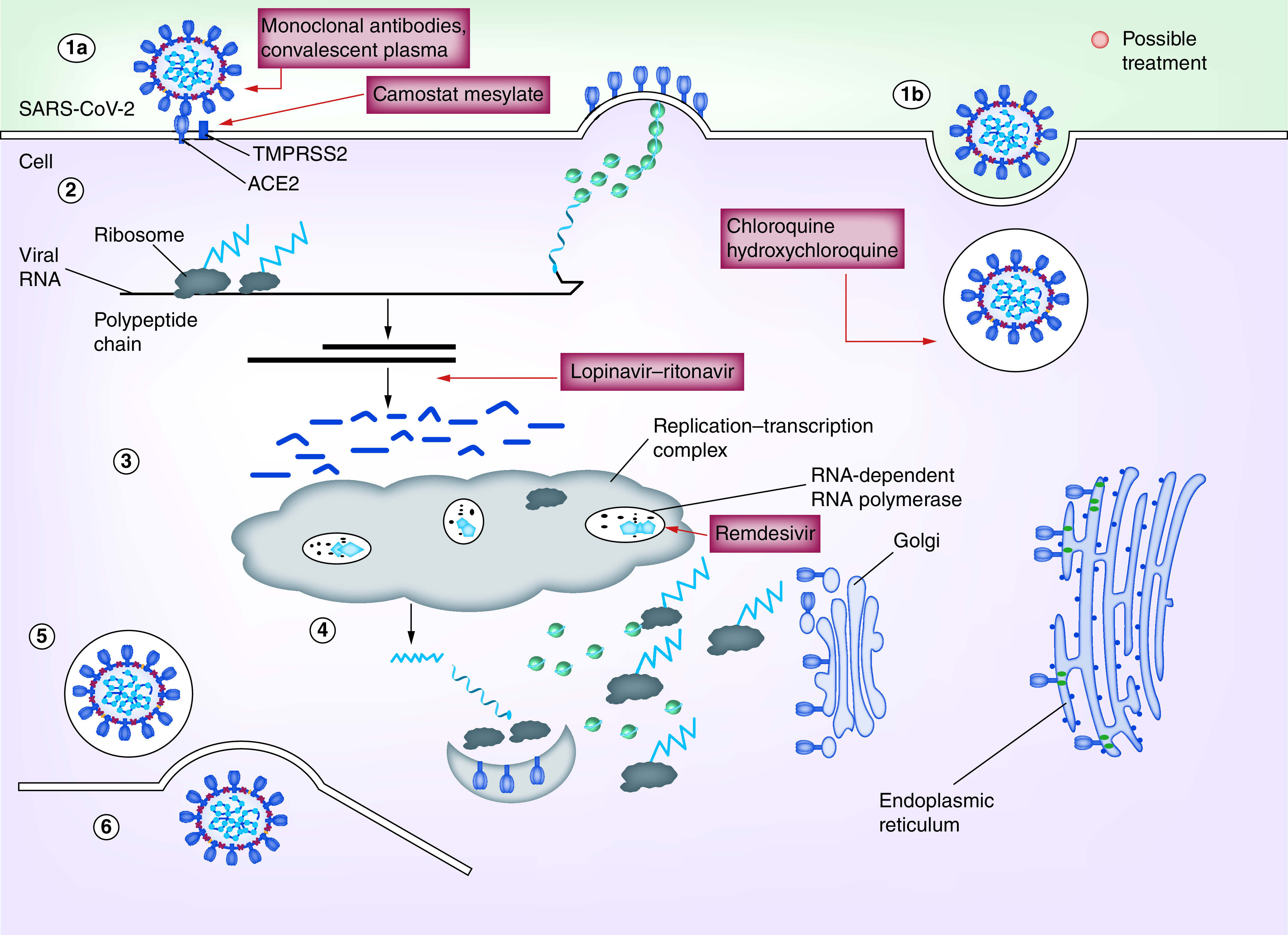
Drug targets to inhibit severe acute respiratory syndrome coronavirus 2 replication. Various therapeutic strategies are being aimed to interfere with viral replication at different points starting from attachment till assembly. **(A1)** Fusion, **(A2)** endocytosis, **(B)** translation, **(C)** proteolysis, **(D)** translation and RNA replication, **(E)** packaging.

All these drugs can be good choices to treat COVID-19 infections. No specific antivirals have yet been reported which may be fully effective against COVID-19. Scientists are working hard to find out the potential drugs but there is a need to do more clinical and preclinical trials of these drugs to test the efficacy and safety in COVID-19 treatment. Different drugs targeting viral replication at different steps have been shown in Figure 1.

### Immunotherapeutic strategies

#### Immunotherapeutic use of the complement system

The complement system is one of the vital components of the innate defence system of the human body which acts as a bridge between innate and adaptive immunity. It consists of more than 35 proteins and regulators. The research on the complement system is progressing rapidly after the establishment of its role in inflammation and a potential therapeutic effect against certain diseases. There are two main types of complement immunotherapy: one in which immune response is enhanced (by using a recombinant form of complement proteins, etc.) while in other type, body's immune responses are suppressed to control the disease by different inhibitors and biomolecules [54,55]. In case of SARS-CoV-mediated acute respiratory distress syndrome, it was observed that the C3 component of complement activation further worsens the disease. As SARS-CoV is closely related to SARS-CoV-2, it can be speculated that the same mechanism could possibly occur in case of COVID-19. Animal models of SARS-CoV also revealed that mice deficient in C3 showed limited respiratory dysfunction [56].

This clearly indicates that inhibition of C3 can be utilized as an effective treatment in case of SARS-CoV-2. *In vivo* infection model studies of AMY-101 (C3 inhibitor) showed that it interferes with IL-6 release [57]. Data on acute respiratory distress syndrome and SARS-CoV-2 is very limited; however, according to a recent report, it was observed that COVID-19 patients displayed a widespread activation of complement proteins, C3a and C5a [58]. All these findings clearly indicate that C3a and C5a inhibitors, as well as other known biomarkers of inflammations can be used to treat COVID-19 patients. More studies need to be conducted to investigate the potential use of the complement system as a therapeutic strategy against SARS-CoV-2.

#### Natural killer cell therapy

Besides boosting antibody responses, enhancing cell mediated immunity is also under consideration. Natural killer cells, also known as K or killer cells, are components of the innate immune system, which play a major role in controlling tumours and rejection of virus-hijacked cells, hence restricting the pace of replication and subsequent tissue damage. Recently, the FDA approved investigational new drug CYNK-001 for Phase I/II clinical studies in COVID-19 patients. Initially, CYNK-001 was developed from placental hematopoietic stem cells and was suggested for the treatment of various haemolytic cancers and solid tumours such as multiple myeloma, myeloid leukaemia and glioblastoma multiforme. Different studies revealed that during viral infection, there is vigorous activation of natural killer cells and CYNK-001 has the potential to express natural cytotoxic receptors as well as activating receptors which bind with viral antigen on infected cells. Due to these functions, it was suggested that CYNK-001 may be used in inhibiting the replication of SARS-CoV-2 and progression of COVID-19 by eradicating the infected cells [19].

#### IL-6 Inhibitors

A couple of recent studies have suggested the effectiveness of tocilizumab in critical patients infected with corona virus. A significant improvement in these patients was observed after its use which ultimately reduced the dependence of patients on artificial oxygen support [20,21]. A likely explanation to this phenomenon is that being IL-6-receptor inhibitor antibody, it breaks the chain of interaction between components of inflammatory cascade, ultimately reducing the effects of cytokine storm. More detailed studies are underway to further elaborate its pros and cons. Several IL-6 inhibitor monoclonal antibodies are under clinical trials (ClinicalTrials.gov identifier NCT04315298 and NCT04331808). Few other anti-inflammatory therapeutic agents, for example, Janus kinase inhibitors, glucocorticoids and chloroquine/hydroxychloroquine have also been suggested for comparatively early recovery of SARS-CoV-2 infected patients [59].

#### mTOR pathway inhibition

In SARS-CoV-2 infection, several altered pathways, for example, ‘IL-17 signalling pathway’, ‘cytokine-mediated signalling pathway’ and ‘defense response to other individuals’ were identified using gene term enrichment analysis [60]. A cytokine storm was also evident, clearly indicating the irregularities and dysfunctionalities in host defense system. These exaggerated innate inflammatory responses and associated dysfunctions in adaptative immune responses are the root cause of tissue damage in COVID-19 infection [61].

Scientists are seeking the novel therapeutic strategies to combat COVID-19 infection. One such novel strategy is to exploit the mTOR for controlling infection. mTOR1, mTOR2 and AMPK are the associated pathways of mTOR [22,62]. The main function of mTOR pathway is to regulate the survival, growth, proliferation and metabolism of the cell. It is also involved in controlling the lipids metabolism, synthesis of proteins, transcription and autophagy as it senses the extracellular as well as intracellular signals [63].

A recent study has speculated that at an early stage of COVID-19, cross-reactive antibodies production against SARS-CoV-2 can be reduced if memory B cells are inhibited selectively, which ultimately reduce the antibody dependant enhancement process, which may contribute toward initiating cytokine storm and make the symptoms worse. mTOR pathway inhibitors, such as rapamycin, have the potential to selectively inhibit the memory B cells leading to a reduction in early-stage crossreactive antibodies against SARS-CoV-2 and resultantly less antibody dependant enhancement [64]. Another study also suggested that sirolimus, an mTOR inhibitor, can be a perfect drug of choice in SARS-CoV-2 infected patients [60]. In addition, the inhibitor also helps in the suppression of potent CD4^+^ effector T cell and promotion of Treg [65,66]. Rapamycin has already been shown to exert antiviral activities against HIV and other viruses like vaccinia and influenza [67–71].

### Vaccination strategies

One of the most effective ways to prevent a disease is vaccination. Vaccine helps the immune system of the body to identify and fight infectious pathogens by inducing acquired immunity. The vaccine contains an inactive or attenuated pathogen, or one of its surface proteins or its toxins, which activate the immune system of the body and keeps us protected from the disease they cause [72]. To control the outbreaks of coronavirus, it is necessary to make a vaccine which helps in reducing the severity, shedding and transmission of the virus.

There are numerous strategies of vaccinations for SARS-CoV-2 such as RNA vaccines, DNA vaccines, Recombinant protein vaccines, live attenuated vaccines, killed/inactivated vaccines and viral vector-based vaccines. In all these vaccines, target is S protein except the live-attenuated and killed vaccine for which the whole virion is the target. Along with many advantages, they have several disadvantages. Against MERS-CoV and SARS-CoV, these strategies have already been tested using animal models [73,74] and many of them are in first clinical-stage trials. Developing a final vaccine after these trials may take several months to many years. S protein has been considered as an ideal target for SARS-CoV and MERS-CoV vaccine. Since it is established that SARS-CoV2 has almost 80% structural similarity with SARS-CoV, S protein is a promising target which can act against both types of viruses [25]. During initial studies of the SARS-CoV vaccine, it was observed that live virus results in lung damage by inducing immunopathological reactions like inflammation and infiltration of eosinophils along with other complications hence worsening the outcome of disease [75,76]. Along with these complications, it is also necessary to examine the effect of the virus during recurrent infection as it is common in SARS-CoV infection. Hence, during vaccine trails for SARS-CoV-2, it is necessary to address these complications and effects to ensure efficacy and safety.

Currently, more than 115 vaccines are under investigation against SARS-CoV-2 in different universities, research institutes, drug companies, biotechnology firms, health organizations and several research groups [26]. Even if the research is progressed at a fast pace, it may take several months to develop the first vaccine in the record time by using one of the vaccine strategies discussed above. Currently, it is impossible to guess which strategy will be faster and more effective. mRNA based vaccines are under clinical trials by Moderna Inc, Curevac is also trying to develop a similar vaccine using this approach. Recombinant protein-based vaccines are also being investigated by Expresssion, iBio, Novavax, Baylor College of Medicine, University of Queensland and Sichuan Clover Biopharmaceuticals (focusing on S protein). Vaxart, Geovax, University of Oxford and Cansino Biologics are creating a viral vector-based vaccine (focusing on S protein). Codagenix with the Serum Institute of India, etc. are focusing to develop live attenuated vaccines while DNA vaccines are under trail by Inovio and Applied DNA Sciences with focus on S protein [73]. For COVID-9, Johnson & Johnson and Sanofi also joined hands to create a vaccine by using adenovirus vector platform and Flublok recombinant influenza virus vaccine respectively [27,77,78]. However, these efforts may take at least several months to develop a vaccine which is readily available for human.

Bacille Calmette-Guérin (BCG) vaccine is also thought to reduce the COVID-19 impact, thus could play an important role against SARS-CoV-2 (Curtis *et al.*). It has a beneficial off-target/nonspecific positive effect on the immune system which may help in an early recovery of COVID-19 patients, as found previously against several respiratory infections [79,80]. In controlled trials, the BCG vaccine reduced the infection severity caused by certain viruses having structural similarities with SARS-CoV-2 [81,82]. Different clinical trials are underway in Australia and Netherland to investigate the effectiveness of BCG vaccine in COVID-19 susceptible individuals especially health workers (ClinicalTrials.gov identifier NCT04327206, NCT04328441, NCT04384549, NCT04348370 and NCT04414267) [83,84]. If successful, use of the BCG vaccine would be a crucial tool against COVID-19 infection and other future pandemics. However, for the time being, there is a need to stick to the recommendations of WHO [85].

### Other therapeutic options

Plasma therapy is another therapeutic strategy used against COVID-19 patients just like SARS-CoV [86] and MERs-CoV [87] in which plasma from recovered patients was injected to infected individuals. Initially, it showed promising results in COVID-19 patients, still further extensive studies are required to investigate if this therapy is worth application keeping in mind complications associated with other plasma proteins. Contini [28] in his research suggested two protease inhibitors for COVID-19 treatment, which have previously been used against HIV infection [28]. The recombinant human monoclonal antibody is one of the other promising therapeutic candidates for infections caused by COVID-19 [29]. For the treatment for SARS-CoV-2, other monoclonal antibodies, already used against SARS-CoV, can be used as an alternative strategy to treat infection [30].

Plants have been used for centuries as therapeutic agents against several diseases, including viral infections. Several medicinal plants have been studied in past against different viral diseases due to the presence of a repository of antiviral metabolites like alkaloids, flavonoids, terpenoids and phenols [88]. Many Chinese scientists have screened several natural products and medicinal plants among which they suggested nine plants and recommended that they might have a role in inhibition of viral replication [12,31].

## Conclusion

This review has presented various strategies which can be used to control COVID-19 infection. COVID-19 pandemic has infected millions of people worldwide and claimed several thousand deaths. Scientists and researches around the globe are working to explore potential therapies against this deadly virus. Different strategies like controlling of replication of the virus, inhibiting the binding of the virus to host cells, use of molecules and compounds to boost innate as well as passive immunity or to screen drugs effective against other viruses can be effective and quick ways to treat COVID-19. Until now there is no approved drug available against SARS-CoV-2 and hundreds of the vaccines and antiviral drugs are under clinical trials which will take several months to be made available in the market. Some antiviral drugs and strategies have shown significant effects *in vitro*, yet there is a need to confirm their efficacy and safety in preclinical and clinical trials. It is hoped that by using these strategies, scientists will develop new, fast, accurate, effective, cheap and safe therapeutic compound against SARS-CoV-2.

## Current scenario & future perspective

COVID-19 emerged at the global scene very recently and rapidly spread across the globe in a couple of months giving scientists very limited time to respond. New drugs and vaccines development usually take years if not decades. The sudden outbreak of COVID-19 caused by a virus, new to the human race, created panic in the scientific community. It was impossible to develop a new drug or vaccine within months or year. Short term approach suggested by scientists for the treatment of COVID-19 patients was to investigate drugs already in practice against other diseases and check their efficacy against SARS-CoV-2. For vaccine development, scientists throughout the world responded rapidly with the help of funding from Govts and industrial sectors and conducting research at a very fast pace. Hence a large number of vaccines are under investigation with some showing promising results.

Luckily SARS-CoV-2 is the sister strain of previously reported SARS-CoV and MERS viruses. Past studies performed against these two strains of coronaviruses are providing a strong baseline for drugs and vaccine development against SARS-CoV-2. Some of the strategies have proven very effective *in vitro* and in animal models. Many have been approved for clinical trials. Hence it is hoped that the human race will be successful in discovering effective drugs for the treatment of severe COVID-19 patients. Besides, some positive news in vaccine development suggest that very soon new vaccines will be available in the market and it will become possible to create a herd immunity against this virus, limiting the spread of this deadly virus and eradicating it from the face of the earth as in case of so many viruses in past.

**Executive summary**

**Background**

- COVID-19 is caused by severe acute respiratory syndrome coronavirus 2 (SARS-CoV-2) also known as 2019 novel coronavirus.
- For SARS-CoV-2 infection, currently, no specific vaccine or antiviral agent is available with clinically proven efficacy. Scientists across the globe are working to develop a vaccine, drug and exploring possible ways to treat this infection.

**Drugs**

- ACE2 receptors inhibitors naturally came across as the major targets of interest for scientific community because of established knowledge that the virus initially binds to a host cell through these receptors. Drugs like baricitinib, ruxolitinib, natural hesperidin and nafamostat mesylate are being investigated to inhibit ACE2 receptors.
- Type II transmembrane serine protease inhibition is also being considered by scientists. Nafamostat mesylate and camostat mesylate have already been approved for type II transmembrane serine protease inhibition.
- Antiviral drugs are usually aimed to stop the replication of virus instead of killing, allowing the body’s own immune response to neutralize the virus.
- The International Clinical Trials Registry Platform of the WHO-approved hundreds of clinical studies to investigate different antiviral drugs already used for treatments of other infections.
- Some of the examples are IFN-α, previously used to treat hepatitis infection. Lopinavir/ritonavir, HIV drugs have displayed some anti coronavirus activity. Similarly, antiviral drug arbidol, favipiravir and darunavir drugs used against HIV and influenza have exhibited some anti-SARS-CoV activity.
- USA FDA approved the antiviral drug remdesivir and ivermectin (an antiparasitic drug) against COVID-19.

**Immunotherapy**

- Although the major focus of therapeutic strategies is on vaccination by using the adaptive immune system, some scientists are suggesting to boost the innate immune system against SARS-CoV-2.
- One of these strategies is to suppress inflammation by blocking the activity of complement protein C3. Complement plays an important role in enhancing inflammation that complicates the disease at later stages leading to severe pneumonia.
- The second strategy is enhancing the function of natural killer cells. A drug like CYNK-001 has the potential to express natural cytotoxic receptors as well as activating receptors which bind with viral antigen on infected cells, creating more sites on virus hijacked cells to be identified by natural killer cells.
- Other Immunotherapeutic approaches like IL-6 and nTOR inhibitors are also under consideration.

**Vaccination**

- Vaccination is the best strategy to prevent a disease by facilitating indigenous immune system of the body to identify and fight against infectious pathogens.
- Various strategies of vaccinations for SARS-CoV-2 such as RNA vaccines, DNA vaccines, recombinant protein vaccines, live attenuated vaccines, killed/inactivated vaccines and viral vector-based vaccines are under investigation.
- Currently, more than 115 vaccines are under investigation. Majority of vaccine strategies are targeting S protein except the live-attenuated and killed vaccine for which the whole virion is the target.

**Other therapeutic options**

- Other therapeutic strategies like plasma therapy, protease inhibitors for COVID-19 treatment, the recombinant human monoclonal antibody and medicinal plants are being studied for their efficacy to control COVID-19 infection.
